# Therapeutic challenges and new therapeutic targets for combined capillary pulmonary hypertension: a review

**DOI:** 10.3389/fmed.2025.1579112

**Published:** 2025-05-02

**Authors:** Ming Lu, Baoguo Wang, Chunyan Rong, Yin Wang, Weihua Zhang

**Affiliations:** Jilin University, Changchun, China

**Keywords:** combined pre-and postcapillary pulmonary hypertension (Cpc-PH), pathophysiological mechanisms, targeted therapy, current medical and surgical treatments, clinical trials, pulmonary vascular resistance (PVR)

## Abstract

With a high frequency and a poor prognosis, combined pre-and post-capillary pulmonary hypertension (Cpc-PH) is a significant subtype of pulmonary hypertension linked to left-sided heart disease (PH-LHD). The complicated pathophysiology of Cpc-PH is primarily characterized by elevated pulmonary venous pressure leading to an increase in retrocapillary pressure, which is followed by elevated pulmonary artery pressure and a marked rise in pulmonary vascular resistance (PVR). There is currently no well-defined treatment plan for Cpc-PH, and there are numerous obstacles to overcome. In patients with Cpc-PH, the effectiveness of targeted medications for pulmonary hypertension is limited and debatable. Recent research has revealed that the prevalence and progression of Cpc-PH may be influenced by genetic factors, metabolic syndrome, oxidative stress, and fibrosis. To help doctors better manage and treat patients with Cpc-PH, this review provides a detailed description of the disease’s epidemiology, pathogenesis, diagnostic techniques, current treatment status, and potential therapeutic targets.

## Introduction

The 2022 European guidelines for the diagnosis and treatment of pulmonary hypertension (PH), the 2022 ESC/ERS Guidelines for the Diagnosis and Treatment of Pulmonary Arterial Hypertension, define PH as the mean pulmonary artery pressure (mPAP) measured by right heart catheterization (RHC) at sea level and at rest ≥20 mmHg, and pulmonary artery wedge pressure (PAWP) > 15 mmHg (1). Compared with the 2015 guidelines, the threshold for mPAP was lowered from 25 mmHg to 20 mmHg, and the threshold for PVR was lowered from 3 WU to 2 WU, which is more conducive to the early detection and diagnosis of patients with PH. Pulmonary arterial hypertension associated with left-sided heart disease (PH-LHD) is the most common form, accounting for approximately 68.5% of PH cases (2). PH-LHD is divided into two subtypes: Isolated postcapillary PH (Ipc-PH): mPAP>20 mmHg, PAWP>15 mmHg, PVR ≤ 2 WU; Combined pre-and post-capillary PH (Cpc-PH): mPAP>20 mmHg, PAWP>15 mmHg, PVR > 2 WU (3). Combined pre-and postcapillary pulmonary hypertension (Cpc-PH) is a specific phenotype of left-sided heart disease-associated pulmonary hypertension (PH-LHD, WHO category 2) characterized by the coexistence of retrocapillary hypertension (increased pulmonary venous pressure) and pulmonary vascular remodeling (precapillary hypertension) due to left-sided heart disease.

**Figure 1:**
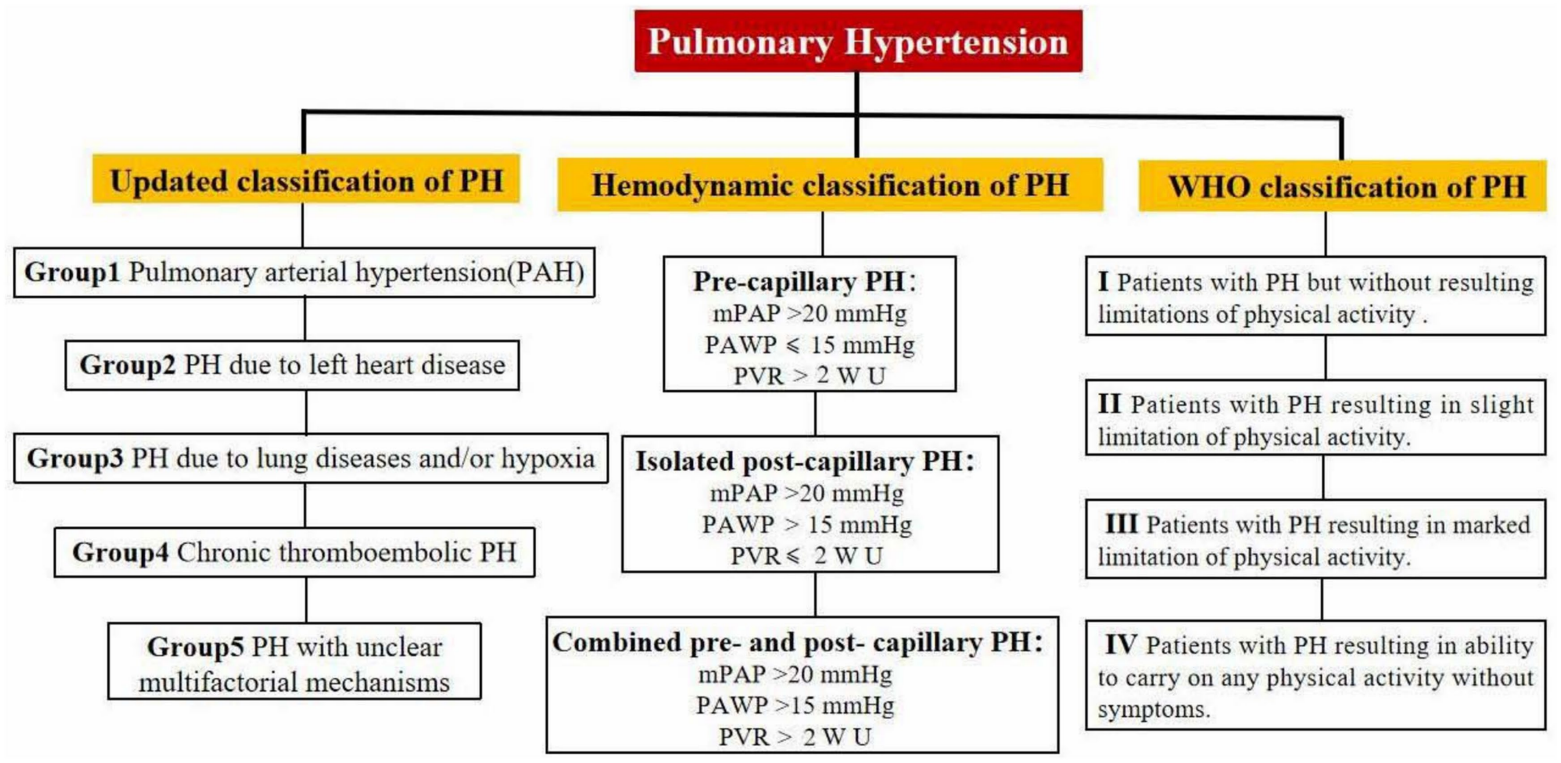
WHO classification, etiological classification, and hemodynamic classification of pulmonary arterial hypertension.

In hemodynamics, Cpc-PH is usually associated with left heart disease or lung disease, which leads to elevated pulmonary venous pressure and subsequently affects pulmonary arterial pressure. In the early stage, the elevation of PAP is mainly due to the passive transmission of left atrial pressure. At this stage, PVR usually remains within the normal range and has not yet caused significant changes in pulmonary vascular structure. As the left atrial pressure continues to rise, changes begin to occur in the pulmonary vascular structure: veins become more permeable, with collagen deposition, leading to thickening of the vessel walls and narrowing of the lumen; capillaries and small arteries, under long-term high-pressure stimulation, develop intimal fibrosis and medial hypertrophy of the pulmonary arteries, ultimately resulting in an increase in PVR. When PVR exceeds 2 WU, Cpc-PH is established. The probability of hospitalization for heart failure or all-cause death increased by 2% for every 1 mmHg increase in mPAP, and by 7% for every 1 WU increase in PVR (4). According to the new definition, the ideal PVR threshold is nearly 2 WU, or 2.2 WU. Combined capillary pulmonary hypertension is difficult to diagnose and treat; a customized treatment strategy must be created, taking into account the effects of lung and left heart disease (Figure 1). This holds significant importance in comprehending the pathophysiological process of pulmonary hypertension and enhancing patient outcomes.

## Epidemiology

About 1% of people worldwide have pulmonary hypertension, and among those 65 and older, the frequency rises to 10%. In developed countries (eg, Europe and the United States), the annual incidence of Cpc-PH is about 5–10 cases per 100,000 people, accounting for 5–10% of all new cases of pulmonary hypertension. The age of onset is more common in the elderly (> 65 years) (1–5), which is associated with a high incidence of left-sided heart disease, and the proportion of women is slightly higher. Hypertension (>70%), diabetes, and obesity (BMI > 30%) are major risk factors in patients with underlying medical conditions, and the incidence of Cpc-PH is higher in patients with valvular disease (eg, mitral regurgitation) and atrial fibrillation. Mortality is significantly higher in patients with Cpc-PH than in PH-LHD alone (2-year survival rate is about 50–60%, 5-year survival rate is about 40–50%, vs. 70–80%), and the risk of death is increased by 20–30% for every 1 WU increase in PVR (3–6). In studies of various types of pulmonary hypertension, mildly elevated mPAP (21–24 mmHg) was significantly associated with increased mortality (3–7). Several studies of patients with systemic sclerosis have found that patients with 24 mmHg ≥ mPAP ≥ 21 mmHg not only have higher mortality but are more likely to progress to pulmonary hypertension (mPAP ≥ 25 mmHg) (3). A total of 5–12% patients with left-sided heart disease (PH-LHD) developed Cpc-PH. According to EU-US and US cohort studies, 1.5–3% of patients with heart failure with reduced ejection fraction (HFrEF) also developed Cpc-PH; and between 2 and 5% of patients with Heart Failure with Preserved Ejection Fraction (HFpEF) developed Cpc-PH (due to pulmonary vascular disease and common metabolic syndrome). Pulmonary hypertension is linked to a twofold increase in mortality in people with both HFrEF and HFpEF. The 3-and 5-year death rates for PH-LHD were 42.3 and 52.6%, respectively, in a cohort study of 4,621 patients with the condition (8). However, all of these PH-LHD clinical cohort studies are from Western nations. From Ipc-PH to Cpc-PH, the annual conversion rate is about 3–6% (9). In certain groups of people with systemic sclerosis, left-sided heart disease and PAH may coexist, increasing the risk of Cpc-PH. The elderly, diabetic patients (30–40%) and patients with chronic kidney disease (GFR < 60 mL/min/1.73 m^2^) are more likely to have mixed pH (10).

China currently lacks representative data on PH-LHD, particularly in the form of long-term follow-up reports. Therefore, multi-center registration studies and the creation of a PH-LHD/Cpc-PH database based on RHC as the standard diagnosis in China are urgently needed.

## Pathophysiological mechanisms

Cpc-PH involves complex pathophysiological mechanisms. The increase of PAWP reflects left atrial dysfunction. This increase in pressure will be transmitted to the pulmonary circulation, leading to the increase of PAP and PVR, and the decrease of pulmonary artery compliance (PAC). It will eventually lead to endothelial dysfunction, pulmonary vascular remodeling and further increase the afterload of the right ventricle.

### Pulmonary vascular remodeling and fibrosis mechanisms

Pulmonary vascular remodeling is the core pathological feature of Cpc-PH, characterized by intimal proliferation, medial hypertrophy, and adventitial fibrosis of the pulmonary arterioles. In the early stages, elevated left ventricular filling pressure leads to impaired pulmonary venous return, increased pulmonary vascular pressure, but no significant structural changes, termed IPC-PH (passive PH) (11). As the disease progresses, structural and functional changes in the pulmonary arteries occur, with thickening and hardening of the vascular walls, narrowing of the lumen, increased resistance, and persistent elevation of pulmonary vascular resistance leading to right heart failure, entering the Cpc-PH phase (12). Patients with Cpc-PH have a higher incidence of right ventricular dysfunction and pulmonary artery remodeling, with significant features of distal pulmonary artery hypertrophic remodeling, fibrosis, and luminal occlusion (13, 14).

Macrophages interact with pulmonary vascular endothelial cells to drive pulmonary vascular remodeling, with an increase in CD68^+^ cells (M2 macrophage subtype) in the adventitial layer, and adventitial fibroblasts may be the primary mediators of their activation and polarization. Fatty acid-binding protein 5 (FABP5) exacerbates pulmonary artery fibrosis by activating the Wnt/*β*-catenin signaling pathway, significantly upregulated in Cpc-PH mouse models, and closely related to pulmonary artery fibrosis. *In vitro* experiments have shown that inhibiting FABP5 (via siRNA or antagonists) can attenuate TGF-β1-induced fibrotic responses. Specific alveolar capillary endothelial cells (Plvap^+^) can transiently activate mesenchymal gene aSMA, affecting extracellular matrix remodeling, vascular integrity, and cell-to-cell interactions (15, 16).

SBFI-26 is a selective FABP5 inhibitor that can inhibit fibrosis by modulating the Wnt/*β*-catenin signaling pathway, significantly suppressing pulmonary artery fibrosis and remodeling in animal models, reducing pulmonary artery wall thickness and collagen deposition. Circular RNA circALMS1 is downregulated in patients with pulmonary arterial hypertension, and its overexpression can inhibit the proliferation and migration of pulmonary microvascular endothelial cells by suppressing the miR-17-3p/YTHDF2 pathway, improving right heart function (17, 18).

### Neurohumoral mechanisms

These include sympathetic overactivation, activation of the renin-angiotensin-aldosterone system (RAAS), inflammatory cell infiltration, and oxidative stress responses. Angiotensin II (Ang II) activates the RhoA/Rho kinase signaling pathway, inhibits myosin light chain phosphatase (MLCP) and endothelial nitric oxide synthase (eNOS) activity, leading to vasoconstriction and endothelial dysfunction (19). The ACE2-Ang (1–7)-Mas axis promotes the release of nitric oxide (NO) and prostaglandins, exerting vasodilatory, anti-proliferative, and anti-inflammatory effects, with enhanced activity improving pulmonary hemodynamics (20). Recombinant human soluble ACE2 (rhACE2) can improve pulmonary artery pressure and reduce oxidative stress (21, 22).

### Left atrial dysfunction

HFpEF, HFrEF, and VHD can lead to elevated left atrial pressure (LAP), increased volume, and subsequent left atrial enlargement, impaired contractility, and interstitial fibrosis as part of the remodeling process (23). Left atrial remodeling weakens its barrier function, passively transmitting pressure to the pulmonary vasculature, causing elevated pulmonary venous pressure and pulmonary congestion (24). Sudden increases in LAP can lead to “alveolar-capillary stress failure,” disrupting the alveolar-capillary barrier and causing pulmonary edema (25). Under the influence of endothelial dysfunction, neurohumoral factors, and inflammatory cell infiltration, persistent changes in LAP lead to structural abnormalities in the pulmonary vasculature and increased pulmonary vascular resistance (26–28).

### Pulmonary vascular endothelial dysfunction

Endothelial cell injury leads to vasoconstriction, inflammatory responses, and fibrosis. Vasoreactive substances such as nitric oxide (NO), prostacyclin (PGI2), and endothelin-1 (ET-1) collectively regulate pulmonary vasodilation and constriction (29). NO is synthesized and released by eNOS, dilating blood vessels through the NO-sGC-cGMP-PKG pathway, inhibiting smooth muscle cell proliferation, and slowing pulmonary vascular remodeling (30). Elevated aldosterone levels can induce oxidative stress, impair ET-B receptor signaling, reduce NO synthesis and bioavailability, and simultaneously, excessive ET-1 production can inhibit eNOS expression, decreasing NO secretion (31, 32).

### Inflammation and chemotaxis mechanisms

CXCL8 (IL-8) and its receptors CXCR1 and CXCR2 are upregulated in pulmonary arterial hypertension, with CXCL8 promoting the recruitment and activation of inflammatory cells through binding to CXCR1/2, exacerbating pulmonary artery inflammation and remodeling. The interaction between CXCL10 and its receptor CXCR3 plays a significant role in the pathogenesis of PH, with CXCL10 inducing chemotaxis of T cells and natural killer cells, participating in pulmonary artery inflammatory responses. The signaling pathways of CXCL12 and its receptors CXCR4 and ACKR3 also play important roles in PH, with the CXCL12-CXCR4 axis not only regulating the migration of inflammatory cells but also promoting the proliferation of PASMCs through the PI3K/Akt signaling pathway (33–36).

### Genetic factors and molecular signaling pathway mechanisms

BMPR-II is a member of the TGF-*β* superfamily, with heterozygous mutations present in 80% of familial PH patients and 20% of sporadic PH patients, making it a key pathogenic gene for primary and hereditary PH (37–39). It influences cell proliferation, differentiation, tissue repair, inflammation, and angiogenesis through Smad1/5/8 and non-Smad signaling pathways. BMP9 gene mutation rates are as high as 6.7% in East Asian populations, with mutations reducing circulating BMP9 levels, weakening vascular anti-apoptotic and anti-damage capabilities, and increasing the risk of idiopathic PH by over 22 times (40–42). The PPARγ-p53 pathway regulates cell proliferation, apoptosis, and inflammatory responses, affecting pulmonary vascular remodeling (43–47). Other gene mutations (such as SMAD1, SMAD4, SMAD9, CAV1, and KCNK3) have also been identified in BMPR-II mutation-negative families (48–50). CLIC4 is highly expressed under stress conditions, promoting BMPR-II internalization and degradation, inhibiting the BMP signaling pathway, and inducing a phenotypic shift in endothelial cells toward anti-apoptotic and proliferative states (51–57).

With the in-depth study of the pathogenesis of Cpc-PH, more and more evidence suggests that mutations in multiple functional genes (such as BMPRII, ALK1, CAV1, TBX4, KCNK3, SMAD8, EIF2AK4, etc.) play an important role in the pathogenesis of the disease. At present, researchers generally believe that the occurrence of PH is similar to tumors, with a “secondary strike” mechanism, where genetic and environmental factors jointly promote the occurrence and development of the disease.

### Gene therapy and molecular signaling pathway mechanisms

**Figure 2:**
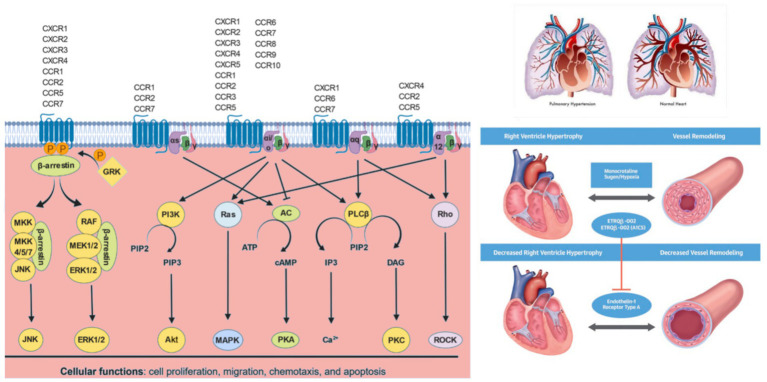
Chemokine signaling pathways: Chemokines bind to their cognate chemokine receptors expressed on different cell types. Upon receptor activation, the G protein dissociates into G*α* and G*β*γ subunits. Depending on the specific Gα subunit(s) to which the chemokine receptor is coupled, various downstream signaling pathways are regulated. Gαs protein activates adenylate cyclase (AC) to produce cAMP, which activates cAMP-dependent PKA. Gαi/o inactivates AC limiting cAMP levels and PKA activity. Gαi/o proteins activate PI3Ks, which converts phosphatidylinositol 4,5-bisphosphate (PIP2) to phosphatidylinositol (3,4,5)-trisphosphate (PIP3), which in turn activates PKB (Akt). Gαi/o and Gα12 proteins activate Ras, which further activates various MAPKs. Gαi/o and Gαq proteins activate PLCβ, which catalyzes PIP2 to IP3 and DAG. IP3 further regulates intracellular free calcium (Ca^2+^) levels, while DAG activates PKC. Gαq and Gα12 proteins also activates Rho family of GTPases (Rho), which further activates Rho-associated PK (ROCK). GPCR kinases (GRK) phosphorylate GPCRs, which enable β-arrestins to bind and internalize GPCRs, which can result in receptor recycling, if receptor phosphorylation is reversed by protein phosphatase 2 (PP2A) or degradation in lysosomes. β-arrestin bound to GPCRs can also activate MAPK pathways such as JNK and ERKs 1 and 2 (ERK1/2).

Experiments based on p53 nanoparticle delivery and AAV vector-mediated delivery of normal BMPR2 genes have shown promising results. Lipid nanoparticles (LNPs) delivering CRISPR-Cas9 systems can repair gene mutations in PASMCs (58–60). Canagliflozin alleviates PH symptoms by activating PPARγ and inhibiting its S225 phosphorylation, showing good protective effects in animal models. Maintaining pulmonary vascular homeostasis by deubiquitination to activate the ALK2-Smad1/5/9-PPARγ axis, with upregulation of BRCC3, can alleviate PH symptoms (61–64) (Figure 2).

## Diagnostic methods

**Figure 3:**
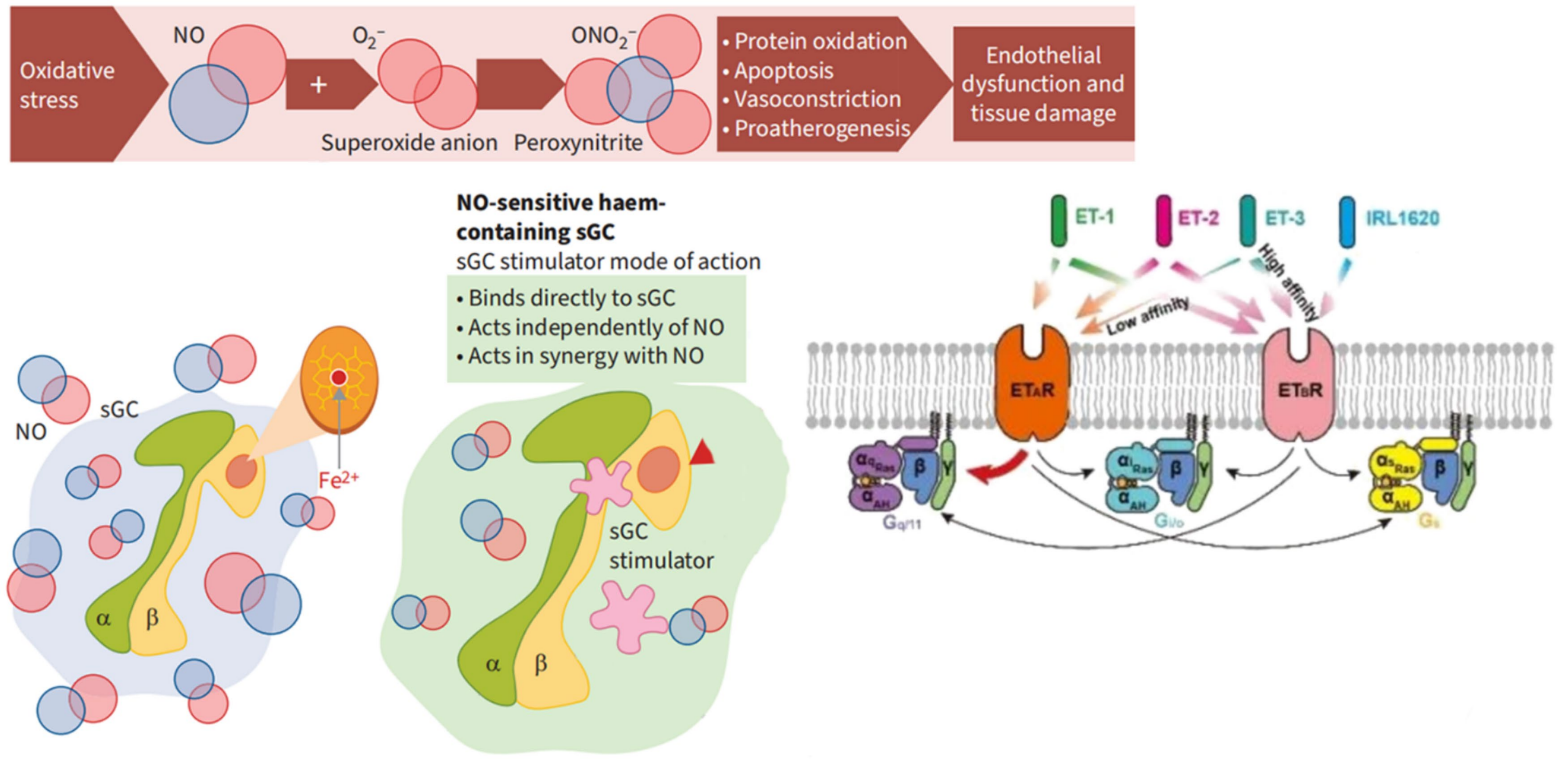
The mechanism of action of soluble guanylate cyclase (sGC) stimulants and activators. α, SGC α subunit; β value, sGC β subunit; cGMP, Cyclic guanosine monophosphate; Fe^2+^, Iron ions oxidize in a + 2 state; NO, Nitric oxide; O^2-^, Superoxide anion; ONO^2-^, Peroxynitrite.

Diagnosis of Cpc-PH is based on a combination of hemodynamic, radiographic, and clinical findings. Right Heart Catheterization (RHC) remains the gold standard for distinguishing PH-LHD/Cpc-PH from other forms of PH, with direct measurement of mPAP, PAWP, PVR, and DPG were directly measured to determine the hemodynamic classification. Diastolic Pressure Gradient (DPG) = Pulmonary Diastolic Blood Pressure-PAWP, DPG ≥ 7 mmHg indicates mixed pulmonary hypertension; Transpulmonary Pressure Gradient (TPG) = mPAP-PAWP, TPG ≥ 12 mmHg may indicate a mixed form. A growing body of data suggests that the ratio of stroke volume to pulmonary artery pulse pressure, a surrogate measure of pulmonary artery compliance (PAC), is associated with poor outcomes in patients with heart failure (65), suggesting that PACs should be given greater attention in the evaluation of early pulmonary vascular disease (66) (Figure 3).

Although right heart catheterization is the gold standard for diagnosing PH, it is invasive. Future research will focus on the development of more precise noninvasive diagnostic techniques, such as the use of advanced imaging techniques (eg, electrocardiogram, magnetic resonance imaging, computed tomography) and biomarker testing, to more accurately assess the hemodynamic status and pulmonary vascular architecture of patients (67). Transthoracic echocardiography (TTE) is the most important screening item used to measure the severity of PH. TTE identifies right ventricular dilation, the presence of valvular regurgitation, and the presence of valvular regurgitation Left ventricular systolic or diastolic dysfunction (68). Ventilation/perfusion scans are also recommended when heart failure (HF) presents with hypercoagulability. Ventilation-perfusion scans remain essential in the clinical workflow, as chronic thromboembolic pulmonary hypertension is unlikely if perfusion is normal (69). Chestx-ray may show cardiomegaly and dilated pulmonary arteries, as well as lung parenchyma or chest wall abnormalities. Chest CT angiography, although considered less sensitive than ventilation-perfusion scanning, may reveal signs of chronic thromboembolic disease, such as filling defects or wedge-shaped or irregular linear opacities due to previous thrombosis. Used to determine the presence of pulmonary artery stenosis or occlusive lesions (70). Serum amino-terminal natriuretic peptide (BNP) levels can be included in risk stratification because they correlate strongly with the severity of pulmonary hypertension and can be used to predict survival (71). The 6MWT reflects the overall exercise tolerance and daily activity ability, and is suitable for assessing functional status and rehabilitation effect. BNP quantifies cardiac stress and the degree of injury, and is suitable for assessing pathophysiological severity and treatment efficacy. The combination of the two can improve the comprehensiveness of the evaluation of patients with Cpc-PH. In patients with PH, elevated BNP reflects right ventricular pressure overload and progression of right heart failure (72, 73).

CardioMEMS is an implantable pressure monitoring system that is implanted into the pulmonary artery via a catheter to monitor PAP, CO, mPAP, PAWP, and others in real time to guide the treatment of heart failure and PH (74). The doctor can remotely monitor the patient’s condition and promptly modify the treatment plan thanks to the data being wirelessly transferred to an external device. This is essential for the evaluation of the condition. In one study, patients treated with CardioMEMS experienced a significant reduction in hospitalization and a significant improvement in quality of life. The CHAMPION study is a multicenter, randomized, controlled trial evaluating CardioMEMS in patients with heart failure (75). The results showed that patients using CardioMEMS had a significant reduction in hospitalization and a significant improvement in quality of life within 6 months. The PULSAR study further explored the use of CardioMEMS in patients with pulmonary hypertension. CardioMEMS was found to significantly improve 6-min walking distance (6MWD) and NT-proBNP levels (76). In a long-term follow-up study, patients treated with CardioMEMS demonstrated sustained improvement in pulmonary artery pressure and cardiac function over 2 years of follow-up, with no device-related serious adverse events (77).

In a study of 208 patients with chronic heart failure, BNP was significantly inversely associated with 6MWD (r = −0.61), and the combined predictive efficacy of the two measures was superior to that of a single measure (78). In patients with PH, 6MWD < 300 m and BNP > 300 pg./mL indicate very high risk and require intensive therapy. Studies have shown that for every 50 m reduction in 6MWD, the risk of death in patients with PH is significantly increased; A decline of >50 m in 6 MWD over 24 weeks is associated with at least a four-fold increase in mortality (79). 6MWT is widely used to evaluate the efficacy of targeted drugs (e.g., Sotatercept), surgery, or exercise rehabilitation. As an example, a mean increase of 40.8 m in 6 MWD followed by pharmacotherapy was accompanied by simultaneous improvement in pulmonary vascular resistance and BNP levels (80). An improvement of 30–50 m in 6MWD is considered the threshold for clinical benefit in patients with PH and is referred to as the minimum clinically important difference (MCID) (81). The peak oxygen uptake cannot be directly measured by 6MWT, and the results are easily affected by factors such as age, gender, and corridor length, so it needs to be comprehensively judged in combination with other indicators. BNP levels were shown to increase significantly with NYHA cardiac function class (Grade I: 186 ± 22 pg./mL; Grade IV: 266 ± 165 picograms/mL) (82). ROC Curve analysis showed that BNP predicted adverse events in patients with chronic heart failure with an area under the curve of 0.914 (sensitivity 0.778, specificity 0.977) (83, 84).

## Current medical and surgical treatments

Based on existing evidence, PH-LHD targeted therapy does not have significant effectsin Cpc-PH patients. Conventional treatment modalities include strengthening the control of underlying diseases, targeted drug therapy, and surgical intervention. Its complexity requires precise hemodynamic assessment and risk stratification, while being alert to the potential risks of targeted drugs. It is recommended to use first-line drugs, including beta blockers, RAAS inhibitors, soluble guanylate cyclase stimulants, and sodium glucose cotransporter 2 inhibitors, in a reasonable combination to improve patient symptoms, delay disease progression, and reduce mortality. For Cpc-PH patients with metabolic syndrome and other diseases, they should also be managed. Although there is limited evidence for the targeted drug application of Cpc-PH, targeted drugs should still be considered in specific situations. The trial outcomes of several medication classes that may be utilized to treat Cpc-PH are reviewed in the sections that follow (Tables 1–3). For Ipc-PH, targeted drug therapy may be harmful because its pulmonary vascular remodeling has not yet occurred, and the increase in pulmonary artery pressure is passive. For Cpc-PH, although targeted drugs may be beneficial for some patients, there is still a lack of clear screening criteria. Future research should focus on screening PH-LHD patients who are sensitive to targeted drugs, especially Cpc-PH patients with high pulmonary artery pressure even after improvement in left heart function (85).

**Table 1:** Endothelin receptor antagonists and PDE 5 inhibitor.

The trial	Drug	Duration	Primary and secondary endpoints	Treatment effects and adverse events
Endothelin receptor antagonists				
MELODY-1	Macitentan	12 weeks	PVR, PAWP, Average right atrial pressure	No significant improvement in PVR, PAWP or Average right atrial pressure, fluid retention, NYHA functional grading worsening.
HEAT	Darusentan	3 weeks	CI, PVR, mPAP, PAWP, Average right atrial pressure	No significant reduction in PVR, mPAP, PAWP or right atrial pressure. The incidence of adverse events in the high-dose group is higher.
EARTH	Darusentan	24 weeks	Cardiac remodeling, clinical status, echocardiography parameters, etc.	No improvement in cardiac remodeling or clinical status, and no obvious benefit was seen in patients with echocardiography suggesting PH.
PDE 5 inhibitor				
RELAX	Sildenafil	24 weeks	6MWD, PVR, NT-proBNP, WHO-FC	6MWD significantly increased by 40.8 meters, PVR significantly decreased, NT-proBNP level decreased significantly, and WHO functional grading improved.
SOVIAC	Sildenafil	12 weeks	mPAP, mPCWP, CO, peak VO₂	Sildenafil leads to worse clinical outcomes such as death and heart failure hospitalization in patients with persistent PH after valve surgery.
Macitentan/Tadalafil combination study	Macitentan/Tadalafil	16 weeks	PVR	The reduction of PVR in the M/T FDC group was significantly higher than that in the monotherapy group. Adverse events leading to discontinuation, serious AEs, and special concerns (anemia, hypotension, and edema) are more common.

**Table 2:** Soluble guanylate cyclase stimulator.

The trial	Type	Study object	Number	Time	Target	Drugs	Endpoints	Conclusion
PATENT-1	Multi-center double blind random placebo	PH	341	12 weeks	NO-sGC-cGMP	Riociguat	6MWD, PVR, NT-proBNP, WHO-FC, TTP, QOL	Improve the exercise capacity and functional indexes of patients with PH, improve the clinically relevant primary and secondary endpoints, delay the occurrence of clinical deterioration, and significantly reduce NT-proBNP.
PATENT-2	Double-blind Random placebo	PH	396	24 months	NO-sGC-cGMP	Riociguat	6MWD, AE, TTP, NT-proBNP, WHO-FC	It is a well-tolerated and effective treatment with sustained improvement in 6MWD and WHO-FC.
CHEST-1	Multi-center open label double blind placebo	CTEPH	243	16 weeks	NO-sGC-cGMP	Riociguat	6MWD, PVR, NT-proBNP, WHO-FC, TTCW, Borg, EQ-5D, LPH	It is effective in improving exercise capacity and cardiac function in patients with inoperable or postoperative recurrent/persistent CTEPH.
CHEST-2	Multi-center opening label double-blind placebo	CTEPH	237	24 months	NO-sGC-cGMP	Riociguat	6MWD, LPH, PVR, EQ-5D, NT-proBNP, WHO-FC, TTCW, Borg,	It can sustainably improve exercise capacity and cardiac function in patients with persistent/recurrent CTEPH after inoperable or PEA, and is clinically and well tolerated.
VICTORIA	Multi-center random double-blind parallel placebo phase III	NYHA II-IV, EF < 45%, BNP ≥ 300 pg./ml, NT-proBNP ≥ 1,000 pg./mL	5,050	10.8 months	sGC-cGMP	Vericiguat	CV Death, First HF, Hospitalization, All-cause Death	Reduce the composite endpoint risk of cardiovascular death or HF hospitalization in patients with HFrEF by reducing recent heart failure exacerbation events.
VITALITY-HFpEF	Multi-center double-blind placebo phase II.b	HFpEF, NYHA II-III, EF ≥ 45%	789	24 weeks	sGC-cGMP	Vericiguat	KCCQ-PLS, 6MWD	There was no improvement in KCCQ-PLS in patients with HFpEF.
DILATE-1	Double blind random placebo control parallel groups stage II.a	LVEF>50%, mPAP ≥ 25 mmHg, PAWP > 15 mmHg	36	30 days	NO-sGC-cGMP	Riociguat	mPAP, SV, SBP, PVR	Riociguat was well tolerated, with 2 mg of riociguat significantly increasing SV and cardiac index CI and decreasing systolic blood pressure, SVR, and RVED area without altering HR, TPG, or PVR
LEPHT	Phase II.b, multi-center double blind random placebo	HFpEF, LVEF ≤ 40%, mPAP ≥ 25 mm Hg	201	16 weeks	NO-sGC-cGMP	Riociguat	mPAP, CI, SV, PVR	Riociguat was well tolerated in patients with PH-1 HFrEF and did not significantly improve mPAP, but significantly improved cardiac index, stroke volume index, and pulmonary vascular resistance in the highest dose group
SOCRATES-PRESERVED	Forward-looking, random double blind placebo phase II.b	HFpEF, LVEF > 45%	477	12 weeks	sGC-cGMP	Vericiguat	NT-proBNP, LAV, KCCQ	Failed to change NT-proBNP, LAV. The KCCQ score improved significantly.
SOCRATES-REDUCED	Multi-center double-blind random placebo control	HFrEF, LVEF≤40%	2,707	12 weeks	sGC-cGMP	Vericiguat	NT-proBNP, Echocardiographic Parameters, All-cause mortality, Cardiac death, Heart failure hospitalization	Vericiguat was well tolerated but did not significantly reduce NT-proBNP levels and did not improve echocardiographic parameters or primary clinical endpoints

CTEPH, Chronic thromboembolic pulmonary hypertension; PH, Pulmonary hypertension; PVR, pulmonary vascular resistance; NT-proBNP, N-terminal brain sodium peptide precursor; EF, Left ventricular ejection fraction; LAV, Left atrial volume; NYHA, New York heart function classification; WHO-FC, WHO heart function classification; QOL, Quality of life; KCCQ-PLS, Kansas City Cardiomyopathy questionnaire activity restricted score; EQ-5D, Quality of life score; LPH, Pulmonary hypertension quality of life; AE, Adverse events; TTP, Time to clinical deterioration; TTCW, Clinical deterioration events; Borg, Changes in dyspnea score; CV Death, cardiovascular death; CI, cardiac index; CO, cardiac output; 6MWD, 6-min walking distance; VO_2_, peak oxygen uptake; LHD, left heart disease; NT-proBNP, N-terminal pro-B-type natriuretic peptide; LVEF, left ventricular ejection fraction; HF, heart failure; Cpc-PH, Combined post-and pre-capillary pulmonary hypertension; HFpEF, heart failure with preserved ejection fraction; LAV, left atrial volume; LVESV, left ventricular end systolic volume; mPAP, mean pulmonary artery pressure; NYHA, New York Heart Association; PH, pulmonary hypertension; SBP, systolic blood pressure; PAWP, pulmonary artery wedge pressure; SPAP, systolic pulmonary artery pressure.

**Table 3:** PGI2 Drugs.

The trial	Drug	Number	Duration	Endpoints	Conclusion
FREEDOM-C	Treprostinil	350	16 weeks	WHO functional class, Borg dyspnea score, dyspnea fatigue index score	Oral Treprostinil for 16 weeks could not significantly improve the activity tolerance, but could significantly change the dyspnea fatigue index and Borg dyspnea classification.
TRIUMPH-I	Treprostinil	235	12 weeks	6MWD, Borg dyspnea score, WHO functional class	Inhaled Treprostinil combined with bosentan or sildenafil treatment significantly improved the 6-min walking distance (6MWD) at 12 weeks.
FIRST	Epoprostenol	471	1 year	Survival, clinical events, congestive heart failure symptoms, 6MWD, quality-of-life measures	CI increased significantly (from 1.81 to 2.61 L/min/m^2^), while PAWP and PVR decreased significantly. However, in patients with heart failure, the survival rate of the Epoprostenol treatment group decreased.
STEP	Iloprost	34	16 weeks	NYHA Functional Class, 6MWD	The combined use of bosentan and iloprost significantly improved the exercise tolerance (6MWD increase) and delayed the disease progression.
GRIPHON	Selexipag	1,156	63 weeks	Survival, clinical events, 6MWD, PVR	The incidence of pulmonary hypertension related events (such as hospitalization, disease progression, death) was significantly reduced, and 6MWD increased by 12.62 meters on average.

### Endothelin receptor antagonists

So far, four types of ERAs have been clinically tested, namely bosentan, anlisentan, Masitentan, and Sitaxentan. Among them, Sitaxentan has been withdrawn from the market worldwide due to fatal liver injury (75). Endothelin-1 (ET-1) participates in vascular constriction and cell proliferation by activating endothelin receptors (ET-A and ET-B) (86). Cochrane studies have shown that using endothelin receptor antagonists for 3–6 months significantly improves patients’ exercise capacity, symptoms, and cardiorespiratory hemodynamic indicators (87). However, it is currently unclear whether these drugs can significantly reduce the mortality rate of patients (88). In PH-LHD, increased expression of endothelin-β1 is associated with disease progression, making it a potential therapeutic target (89). However, ERA has limited efficacy in PH-LHD and is associated with a high risk of adverse reactions (90). Common adverse events include headaches, anemia, and edema, which are usually dose-dependent (91). Bosentan may cause liver dysfunction, but it can usually be restored after reduction or discontinuation of the medication (92). New ERAs, such as Masitentan, have reduced the incidence of these adverse reactions by optimizing their pharmacokinetic properties (93). In the study of patients with advanced HFrEF, bosentan failed to improve NYHA functional grading, systolic pulmonary artery pressure (PAP), tricuspid regurgitation velocity, etc., causing fluid retention and peripheral edema, and increasing the hospitalization rate of heart failure (94). Macitentan did not significantly improve PVR, PAWP, or mRAP after 12 weeks of treatment for Cpc-PH patients, according to the MELODY-1 trial; however, the difference was not statistically significant (95). Although daluxostane therapy did not significantly lower PAWP, mPAP, PVR, or RAP, it did improve cardiac index in HF patients after 3 weeks in the HEAT study. In the high-dose group, adverse events were more common. The EARTH trial also showed that, even in individuals with echocardiographic evidence of PH, Darusentan treatment for 24 weeks did not improve clinical status or cardiac remodeling in patients with chronic HF. In clinical trials of PH-LHD, ERA has generally demonstrated a high incidence of side events; several trials were prematurely stopped because of safety concerns. Therefore, currently ERA is not recommended for the treatment of PH-LHD/Cpc-PH.

### Phosphodiesterase (PDE 5 inhibitor)

PDE5 inhibitors can improve PH and right ventricular function by inhibiting PDE5 enzymes, leading to vasodilation. A Cochrane review suggests that PDE5 inhibitor treatment can increase patients’ average 6-min walking distance by 48 meters, while improving their functional level and reducing the risk of hospitalization associated with PAH. The AMBITION trial showed that the initial combination of Ambrisentan (ERA) and Tadalafil (PDE5 inhibitor) significantly reduced the risk of clinical failure. In HFrEF patients, multiple early small-scale studies have shown that sildenafil significantly reduces pulmonary artery systolic pressure (PASP), mPAP, PVR, 6MWT, and improves right ventricular function (96, 97). Sildenafil did not increase exercise capacity, left ventricular mass, or clinical composite endpoints (death, cardiac/renal hospitalization, increased heart failure symptoms, etc.) as compared to placebo treatment in a large randomized controlled trial (*n* = 216). The sildenafil group saw a modest increase in vascular adverse events, including headache, flushing, and hypotension, however these were not statistically significant (98, 99). The efficacy of PDE5 inhibitors in HFpEF patients is not yet clear, especially in patients without right ventricular dysfunction. Right ventricular dysfunction may be an important predictor of the benefit of PDE5 inhibitor therapy (98). HFpEF patients with concomitant right ventricular failure may benefit from PDE5 inhibitor therapy, such as reducing right atrial pressure and improving right heart function. HFpEF patients without right ventricular dysfunction did not show significant benefits (100). Multiple meta-analyses have summarized the efficacy of PDE5 inhibitors (including sildenafil) in PH-LHD. In HFrEF patients, PDE5 inhibitors significantly improved mPAP PVR, LVEF, physical ability and quality of life (101, 102). PDE5 inhibitors, such as sildenafil, have shown certain clinical benefits in HFrEF patients, particularly in improving hemodynamics and exercise capacity (103). In the SOVIAC study, sildenafil even led to worse clinical outcomes such as death and hospitalization for heart failure in patients with persistent PH after valve surgery (104). The TRITON study evaluated the efficacy of triple combination therapy (Masitentan, Tadalafil, and Selexipag) versus dual combination therapy (Masitentan and Tadalafil) in newly diagnosed, untreated PH patients, and the results showed no significant difference in the primary endpoint of PVR between the two. A cohort analysis of the Spanish PH registry investigated the predictive factors of PDE5 inhibitor treatment response. The results showed that male gender, diagnosis of portal pulmonary hypertension (PoPH), or HIV-PAH were independent predictors of favorable response to PDE5 inhibitors (105). And carbon monoxide dispersibility (DLco) ≤ 40% of the predicted value is associated with adverse reactions. For patients who have received PDE5 inhibitors but have not achieved the desired clinical efficacy, conversion to soluble guanylate cyclase (sGC) stimulants may be considered.

### Soluble guanyl cyclase stimulators

Soluble guanyl cyclase stimulators promote vasodilation by enhancing the production of cyclic guanosine monophosphate (cGMP), complementing the PDE5 inhibitor (which works by reducing cGMP degradation) mechanism, and have received extensive attention in the field of heart failure (HF) and pulmonary hypertension (PH) in recent years. Vericiguat was well tolerated in patients with HFrEF in the SOCRATES-REDUCED study, but did not significantly reduce NT-proBNP levels or improve echocardiographic parameters (106–109). In the SOCRATES-PRESERVED study, vericiguat did not significantly reduce NT-proBNP levels, but it was well tolerated and associated with improved quality of life (110). In the VICTORIA study, vericiguat significantly reduced the composite endpoint of cardiovascular death or hospitalization for heart failure in patients with symptomatic worsening HFrEF (111). In the LEPHT trial, riociguat was well tolerated in patients with HFrEF, significantly improving cardiac index, stroke volume index, and PVR in the high-dose group (112). In the DILATE-1 trial, Riociguat improved stroke volume and systolic blood pressure, but had no significant effect on mPAP and PVR (113). The LEPHT study evaluated the safety and efficacy of riociguat in patients with HFrEF and pulmonary hypertension (PH) (114). The study showed that after 16 weeks of treatment with riociguat (2 mg, TID), patients had an increase in cardiac index and a significant decrease in pulmonary vascular resistance (PVR) and systemic vascular resistance (SVR) compared with the placebo group. The DILATE-1 study was in patients with HFpEF and PH (LVEF > 50%, mPAP ≥ 25 mmHg, PAWP > 15 mmHg). The results showed that after 6 h of treatment, the levociguat 2 mg group showed improvements in stroke volume and right ventricular end-diastolic area, although there was no significant decrease in mean pulmonary artery pressure (mPAP) (115). The COMPERA 2.0 study evaluated the safety and efficacy of riociguat in patients with pulmonary hypertension and cardiometabolic comorbidities. The PASSION study is planned to evaluate the effects of riociguat on exercise tolerance, cardiac function indexes, and WHO functional classification in patients with heart failure and pulmonary hypertension. Riociguat has shown potential benefits in patients with HF-related Pulmonary Hypertension in some studies, such as improved PVR, SVR, 6MWD, suggesting that it may have a positive effect on exercise tolerance (116). In a study of 61 patients with pulmonary hypertension (PH) who had an inadequate response to PDE-5 inhibitors, switching to riociguat resulted in an increase in 6 MWD at 24 weeks, an improvement in WHO grade, and a decrease in NT-proBNP levels (117, 118). In patients with heart failure, vericiguat has shown significant clinical benefit in patients with HFrEF, particularly in reducing the risk of cardiovascular events, but has limited effect in patients with HFpEF (119–121). Higher doses of riociguat improve PVR and systemic vascular resistance in patients with Cpc-PH, but have limited efficacy in patients with Ipc-PH (122, 123). The study, which included data from PATENT-1, PATENT-2, PATENT PLUS and REPLACE, showed that the incidence and severity of adverse events were similar to those in the placebo group in the riociguat group (124).

### PGI2 drugs

Prostacyclin is mainly produced by endothelial cells and has vasodilation, antithrombotic and antiproliferative effects, which can improve hemodynamics and cardiac function. Prostacyclin drugs include prostacyclin analogs (such as Epoprostenol, Treprostinil, Iloprost, Beraprost) and prostacyclin IP receptor agonists (such as Selexipag). Eprostol works by reducing PVR and improving right ventricular function, but long-term use may activate harmful neurohormonal systems (such as the renin-angiotensin system), leading to worsening of heart failure. In the FIRST trial, although epprostol significantly improved cardiac index, pulmonary wedge pressure (PAWP) and systemic vascular resistance (SVR), the trial was terminated early due to the decline in patient survival. Treprolinil is a novel prostacyclin analog that may improve metabolism and cardiac function by activating the AMPK pathway in skeletal muscle and right ventricle. In animal models, Trepronnier improved metabolic syndrome and reduced pulmonary arterial pressure, showing potential to prevent the development of heart failure (HFpEF) with ejaculation fractions. Unlike heart failure (HFrEF), which has reduced ejection fraction, HFpEF is often accompanied by metabolic syndrome and diabetes, and the beneficial effects of prostacyclin analogs on metabolism and pulmonary blood vessels make it possible that it is more suitable for the treatment of PH-HFpEF. In patients with Cpc-PH, targeted pulmonary hypertension therapy (such as phosphodiesterase 5 inhibitors and endothelin receptor antagonists) fails to improve symptoms, but instead increases morbidity and death Rate. A meta-analysis showed that although PH-targeted therapy may improve exercise capacity in patients with left heart disease (LHD), the risk of adverse events is higher. A meta-analysis showed that although pH targeted therapy can improve the exercise ability of patients with left heart disease (LHD), the risk of adverse events is higher, but the efficacy of human pH HFPEF patients still needs further clinical trials to verify, and the treatment strategy of skeletal muscle and right ventricular AMPK pathway may be a potential direction for the prevention of PH-LHD (125–131).

Ralinepag, as a novel prostacyclin receptor (IP receptor) agonist, exhibits significant vasodilation effects, which can effectively reduce PVR while inhibiting vascular smooth muscle cell proliferation and platelet aggregation. Its drug has a half-life of up to 24 h, which gives it a potential advantage in the treatment of PH. In Phase II clinical trials, Ralinepag has shown good efficacy, and common adverse reactions include headache, nausea and diarrhea. At present, the Phase III clinical trial of Ralinepag is being promoted, and the preliminary long-term data of the ADVANCE EXTENSION study have been released at relevant academic conferences (132). At present, prostacyclin drugs have limited their widespread use in clinical practice due to their poor stability and many side effects. Therefore, the development of novel prostacyclin drugs with higher receptor selectivity, fewer side effects, more stable, easier to preserve and more easily accepted by patients has become the research direction (133).

### Sotatercep

Sotatercept is a fusion protein of the type IIA activin receptor (ActRIIA). Results from the PULSAR study show that Sotatercept significantly outperforms placebo in reducing PVR and improving 6MWD, as well as in secondary endpoints such as NT-proBNP levels, WHO functional classification, and clinical worsening events. Results from the STELLAR study indicate that after 24 weeks of treatment, patients in the Sotatercept group saw an increase of 40.8 meters in 6MWD compared to a mere 1.0 - meter increase in the placebo group. Other secondary endpoints, including reductions in NT-proBNP levels, improvements in WHO functional classification, and decreases in PVR, were also significantly better in the Sotatercept group. With a median follow - up of 32.7 weeks, Sotatercept reduced the risk of clinical worsening or death by 84%. Additionally, Sotatercept improved right - heart function by lowering mPAP and reducing the workload on the right side of the heart. In terms of safety, adverse events in the Sotatercept group mainly included capillary dilation, epistaxis, gingival bleeding, and thrombocytopenia, but most of these events were mild. In the *post-hoc* analysis of the STELLAR study, Sotatercept was found to reduce the size of the right side of the heart and improve right - ventricular function and hemodynamic parameters after 24 weeks of treatment. Ongoing trials such as SOTERIA, HYPERION, ZENITH, CADENCE, MK - 7962-020, and MOONBEAM will further explore the long - term safety and effectiveness of Sotatercept in diverse patient populations, providing more comprehensive clinical evidence for the future treatment of pulmonary arterial hypertension. Compared with traditional small - molecule compounds, Sotatercept has higher efficacy and fewer adverse effects. By rebalancing the BMPR2 signaling pathway and inhibiting the Smad2/3 signaling pathway, Sotatercept reduces the proliferation of pulmonary vascular smooth muscle cells and collagen deposition, thereby reversing pulmonary vascular remodeling. This goal is not achievable by most currently available targeted drugs. Moreover, Sotatercept’s dosing schedule of once every 3 weeks and its subcutaneous injection route are more convenient than those of traditional medications (Table 4). With its unique mechanism of action and significant clinical benefits, Sotatercept offers a new treatment option for Cpc-PH patients and has the potential to transform the current treatment landscape (134–138).

**Table 4:** Sotatercept.

Trial name	Clinical Trials.gov ID	Type	Trial population	Primary endpoint	Secondary endpoints	Trial results	Number	Duration
PULSAR	NCT04811092	Randomized, double-blind, placebo-controlled, Phase III	Newly diagnosed intermediate- and high-risk PAH patients	PVR	6MWD, NT-proBNP levels, WHO FC, Clinical worsening events	Sotatercept significantly reduced PVR, increased 6MWD, improved NT-proBNP and WHO functional classification	106	24 weeks
STELLAR	NCT04896008	Randomized, double-blind, placebo-controlled, Phase III	WHO functional class III or IV high-risk PAH patients	Change in 6MWD at 24 weeks	Multicomponent improvement, PVR, NT-proBNP, WHO functional classification, clinical worsening events	Sotatercept significantly increased 6MWD, reduced PVR, improved NT-proBNP and WHO functional classification	323	24 weeks
HYPERION	NCT04811092	Randomized, double-blind, placebo-controlled, Phase III	Newly diagnosed intermediate- and high-risk PAH patients	Time to clinical worsening	6MWD, NT-proBNP levels, WHO functional classification	Terminated early due to significant interim results	662	Estimated completion in January 2030
ZENITH	NCT04896008	Randomized, double-blind, placebo-controlled, Phase III	WHO FC III or IV high-risk PAH patients	Time to clinical worsening	Overall survival, transplant-free survival	Interim analysis showed significant reduction in clinical worsening events, terminated early	166	Estimated completion in November 2025
SOTERIA	NCT04796337	Open-label, long-term extension study	Adult PAH patients who completed a sotatercept parent study	Number of adverse events and time to treatment discontinuation	Long-term safety, tolerability, and efficacy	Ongoing, expected to enroll 700 participants	700	Long-term (ongoing)
CADENCE	NCT04945460	Randomized, double-blind, placebo-controlled, Phase II	PH patients with heart failure and preserved ejection fraction	Change in PVR	Change in 6MWD	Ongoing, expected to enroll 150 participants	150	Estimated completion in February 2027
MK-7962-020	NCT05818137	Non-randomized, open-label, Phase III	Japanese PAH patients	Efficacy and safety	–	Ongoing, expected to enroll 35 participants	35	Estimated completion in August 2025
MOONBEAM	NCT05587712	Open-label, Phase II	PAH children aged 1–18 years	Safety, tolerability, pharmacokinetics, and pharmacodynamics	–	Ongoing, expected to enroll 42 children	42	Estimated completion in 2028

## Ongoing clinical trials

Many patients with cardiovascular diseases often have metabolic syndrome (MS), such as obesity, dyslipidemia, insulin resistance, and diabetes (Table 5). MS can induce systemic inflammatory responses, and inflammatory factor infiltration and imbalance in immunomodulation are key pathogenic drivers of vascular remodeling. Therefore, immunotherapy may become a new therapeutic strategy for PH-LHD/Cpc-PH (139–142) (Table 6).

**Table 5:** Ongoing clinical trials.

Drug name	Drug type	Mechanism of action	Development stage
TX45	Long-acting Fc-Relaxin fusion protein	Improves pulmonary hemodynamics and left ventricular function	Phase Ib: Single intravenous infusion significantly reduced PCWP by 17.9% and PVR by 30%. Phase II APEX Trial: Ongoing, with topline data expected in 2026.
Seralutinib	Tyrosine kinase inhibitor	Inhibits relevant signaling pathways to improve pulmonary arterial hypertension	TORREY trial (phase II trial): PVR decreased by 14% (*p* = 0.0310), and in patients with more severe symptoms, PVR decreased by 21% (*p* = 0.0427) PROSERA trial (Phase III trial) is currently underway
Imatinib	Tyrosine kinase inhibitor	Non-vasodilator, mechanism not fully elucidated	Inhalation formulation undergoing Phase II and III clinical trials.
Macitentan	Endothelin receptor antagonist	Blocks endothelin signaling pathways, reducing pulmonary arterial pressure	Approved, better efficacy when used in combination with Tadalafil.
MK-5475	Soluble guanylate cyclase activator	Activates guanylate cyclase, increases cGMP levels, and relaxes vascular smooth muscle	INSIGNIA-PAH Phase II clinical trial results published in September 2024.
Inhaled Treprostinil	Prostacyclin analog	Vasodilation, improvement of pulmonary arterial hypertension	*Post-hoc* analysis and extension studies of the INCREASE trial show improved survival in patients with IPF and pulmonary arterial hypertension.
MRE-269	Selective IP receptor agonist	Main metabolite of Selexipag, activates IP receptors to increase intracellular cAMP levels, leading to pulmonary vasodilation	Under investigation.
T26A	Prostaglandin transporter (PGT) inhibitor	Reduces intracellular concentration of PGE2, targeting pulmonary vasodilation	Under investigation.
MN-08	Nitrate ester derivatives of memantine	Antagonism of N-methyl-D-aspartate (NMDA) receptors and release of NO	Approved for clinical trials by China’s NMPA in January 2025, Phase II clinical trial to commence soon.
KER-012	ACVR2B ligand trapper	Blocks signaling of TGF-β superfamily members, reversing pulmonary vascular remodeling	Ongoing Phase II clinical trial.
Recombinant MPB9	Recombinant protein	Targets BMP9, modulates bone morphogenetic protein	In development stage.
Letrozole, Tamoxifen, DHEA	Sex hormones	Regulates sex hormone levels, potentially beneficial for PH	Under investigation.
Metformin, Trimetazidine	Metabolic targeting drugs	Modulates metabolic processes, potentially beneficial for PH	Under investigation.
Canagliflozin	SGLT-2 inhibitor	Inhibits SGLT-2 receptor, reduces right ventricular systolic pressure, improves PH	Under investigation.
Empagliflozin	SGLT-2 inhibitor	Inhibits SGLT-2 receptor	EMBRACE-HF study showed a reduction in pulmonary artery diastolic pressure by 1.7 mmHg compared to placebo at Week 12.
Celastramycin	Benzoylpyrrole compound	Reduces secretion of inflammatory factors and reactive oxygen species, targets anti-proliferative effects on PH-PASMC	Not yet in clinical trials, but animal experiments have shown potential for improving symptoms of pulmonary arterial hypertension.

**Table 6:** Studies uncovering potential therapeutic targets.

Author	Title	Result	Date	Potential therapeutic target
Hou et al. (143)	Targeting Fibroblast Activation Protein for Molecular Imaging of Fibrotic Remodeling in Pulmonary Arterial Hypertension	18F-FAPI PET/CT imaging is feasible for visualizing the remodeling of the PA and the RV in PAH. Although it offers promise for assessing disease-related changes, its role in evaluating disease severity and monitoring therapeutic efficacy requires further investigation.	2025	fibroblast activation protein inhibitor (FAPI)
Zhang et al. (144)	Endogenous hydrogen sulfide persulfidates endothelin type A receptor to inhibit pulmonary arterial smooth muscle cell proliferation	Endogenous H2S persulfidated ETAR at Cys69 to inhibit the binding of ET-1 to ETAR, subsequently suppressed PASMC proliferation, and antagonized pulmonary vascular structural remodeling.	2025	ETAR over sulfurization modification
Li et al. (63)	Genetic recording of transient endothelial activation in distinct alveolar capillary cells during pulmonary fibrosis	Transient EndoMT activation of specific endothelial cells (Plvap+) during pulmonary arterial hypertension and pulmonary fibrosis may affect extracellular matrix remodeling and vascular integrity.	2024	EndoMT
Shen et al. (145)	BRCC3 Regulation of ALK2 in vascular smooth muscle cells implication in pulmonary hypertension	BRCC3 regulates the BMP signaling pathway and maintains pulmonary vascular homeostasis by deubiquitinating K472 and K475 sites of ALK2.BRCC3 deficiency exacerbates pulmonary arterial hypertension, and PPARγ agonists can partially alleviate its effects.	2024	BRCC3-ALK2-Smad1/5/9-PPARy
Harvey et al. (146)	Lysosomal dysfunction and inflammatory sterol metabolism in pulmonary arterial hypertension	NCOA7 regulates lysosome function and cholesterol metabolism, inhibits endothelial cell immune activation, and alleviates pulmonary arterial hypertension. Small molecule compound 958ami can activate NCOA7 and improve the pathological phenotype of PAH model.	2025	NCOA7

## Potential new therapeutic targets

Stem cell therapy, as an emerging therapeutic approach, has attracted much attention for its ability to self-renew, proliferate and differentiate into a variety of specific cells. Stem cells commonly used to treat pulmonary hypertension (PH) include endothelial progenitor cells (EPCs) and mesenchymal stem cells (MSCs). Among them, EPCs are oligona stem cells that tend to differentiate into endothelial cells (EC), but may promote the occurrence and development of PH in a pathological state. For example, EPCs in hypoxic newborn calves showed stronger migration and ductal capacity. To correct the defects of EPCs, cell infusion becomes a direct approach. However, in PH progression, EPCs may reactivate their hematopoietic tendencies by abnormally expressing hematopoietic transcription factors, resulting in excessive infiltration of immune cells. Liang et al. successfully blocked the EHT process and reversed the decline in EPCs levels using the endothelial-hematopoietic transition (EHT) inhibitor Runx1 in a single-dose monoclonal toxin (MCT). In addition, erythropoietin (EPO) has also been shown to have vascular protection effects, which restores the number of circulating EPCs and reverses vascular remodeling by promoting the expression of heme oxygenase-1 (HO-1). Mesenchymal stem cells (MSCs) are pluripotent stem cells that tend to differentiate into mesenchymal cells such as osteoblasts, adipocytes, chondrocytes and myocytes. MSCs show significant potential in the treatment of PH, especially in rat models induced by chronic hypoxia and single-dose monoclonal toxin (SuHx), where MSCs are able to reverse collagen deposition and reduce peripulmonary vasculo-hemophilia factors (vWF) and *α*-smooth muscle actin (α-SMA). In addition, MSCs also improve PH symptoms by regulating intestinal flora. Studies have shown that both SuHx and MCT can destroy the homeostasis of the intestinal microbiota in mice, while MSCs treatment can restore the anti-inflammatory bacterial level and improve the immune-regulating functional bacterial population (147–151).

### Progress in interventional treatment

An emerging interventional therapy technique called pulmonary artery denervation (PADN), which is based on catheter ablation, reduces pulmonary artery resistance, improves right heart function, and delays pulmonary artery disease by inhibiting sympathetic nerve activity by ablation of nerve-intensive areas in or near the pulmonary artery. High pressure (PH) progress. Clinical research and animal experimentation have both produced positive outcomes. According to recent research, PADN can reverse the downregulation of *β*-adrenergic receptors and the overexpression of *α*-adrenergic receptors in rat lung tissues in the preclinical model of pulmonary hypertension-left heart disease (PH-LHD). PADN significantly decreased mean pulmonary arterial pressure (mPAP) and 6-min walking distance in a phase II trial of mixed etiology PH, including PH-LHD. In addition, multi-center trials for Cpc-PH also showed that PADN can reduce systolic and diastolic pulmonary artery pressure, increase cardiac output and 6-min walking distance, while significantly reducing clinical deterioration and hospitalization. Rate. A clinical study in 98 patients with Cpc-PH further confirmed that PADN significantly reduces pulmonary vascular resistance (PVR) and improves exercise tolerance. Although PADN shows good prospects in different types of PH, further studies are needed to evaluate its long-term safety and clinical outcomes (152–155).

In the management of heart failure, ARB is one of the standard treatment drugs, which can indirectly have a positive effect on Cpc-PH by reducing cardiac load and improving cardiac function. In the PADN-5 study, the efficacy of PADN combined with standard drug therapy for heart failure (including ARB) was evaluated in Cpc-PH patients, with significant effects in reducing the incidence of clinical deterioration, improving hemodynamic indicators (such as PVR, PAWP), and enhancing patient exercise endurance. Although there have been studies exploring the potential role of ARB in the treatment of heart failure and related pulmonary arterial hypertension (PH), there is currently no clear clinical research directly confirming the effectiveness and safety of ARB combined with targeted drugs in the treatment of post capillary pulmonary hypertension (Cpc-PH).

In the management of Cpc-PH patients, it is crucial to expand the range of management strategies. A comprehensive assessment should be conducted for each patient, including medical history, physical examination, imaging studies, and hemodynamic monitoring. Existing risk stratification models should be used to rigorously assess the risk level of each patient. A variety of treatment options, such as pharmacological therapy and surgical interventions, should be tailored to the individual patient’s condition. Surgical interventions include pulmonary endarterectomy (PEA), atrial septostomy, pulmonary balloon angioplasty (PBAV), atrial septal defect closure, ventricular septal defect closure, patent ductus arteriosus closure, and lung transplantation. For patients with more complex conditions, emerging treatment technologies, such as PADN or stem cell and other biological therapies, can be explored. A multidisciplinary team should be organized to jointly participate in the diagnosis and treatment process of the patient. By strengthening follow-up, the patient’s condition changes can be monitored in a timely manner, and the treatment plan can be adjusted accordingly. Through this comprehensive management strategy, we can better address the complexity of Cpc-PH and provide more comprehensive treatment support for patients.

## Future

The most prevalent kind of PH is Cpc-PH, and unfavorable prognoses rise in tandem with the patient population’s increased risk of morbidity and mortality. Although there are clear similarities to PH in many areas, the current guidelines do not advocate targets for PH because there are currently no clinical trials that can demonstrate the safety and efficacy of these targeted medications in patients with Cpc-PH. In order to identify novel therapeutic targets, create targeted treatment plans for Cpc-PH, and implement evidence-based strategies to keep HF patients from developing PH, we must thereby expand our understanding of the pathophysiology, etiology, and genetic components of the condition. In order to make sure that treatment approaches can do more than just support physiological processes, we will conduct patient-centered clinical trials as new treatments are developed. These trials should assess the beneficial effects of treatment on patient survival, readmission, and quality of life. Significant clinical improvement can also result from recovery.

## Data Availability

The raw data supporting the conclusions of this article will be made available by the authors, without undue reservation.
